# Markers of schizophrenia at the prosody/pragmatics interface. Evidence from corpora of spontaneous speech interactions

**DOI:** 10.3389/fpsyg.2023.1233176

**Published:** 2023-10-12

**Authors:** Valentina Saccone, Simona Trillocco, Massimo Moneglia

**Affiliations:** LABLITA Laboratory, Department of “Lettere e Filosofia”, University of Florence, Florence, Italy

**Keywords:** schizophrenic speech, prosodic-pragmatic correlates, information structure, prominence, disfluency

## Abstract

The speech of individuals with schizophrenia exhibits atypical prosody and pragmatic dysfunctions, producing monotony. The paper presents the outcomes of corpus-based research on the prosodic features of the pathology as they manifest in real-life spontaneous interactions. The research relies on a corpus of schizophrenic speech recorded during psychiatric interviews (CIPPS) compared to a sampling of non-pathological speech derived from the LABLITA corpus of spoken Italian, which has been selected according to comparability requirements. Corpora has been intensively analyzed in the Language into Act Theory (L-AcT) frame, which links prosodic cues and pragmatic values. A cluster of linguistic parameters marked by prosody has been considered: utterance boundaries, information structure, speech disfluency, and prosodic prominence. The speech flow of patients turns out to be organized into small chunks of information that are shorter and scarcely structured, with an atypical proportion of post-nuclear information units (Appendix). It is pervasively scattered with silences, especially with long pauses between utterances and long silences at turn-taking. Fluency is hindered by retracing phenomena that characterize complex information structures. The acoustic parameters that give rise to prosodic prominence (f0 mean, f0 standard deviation, spectral emphasis, and intensity variation) have been measured considering the pragmatic roles of the prosodic units, distinguishing prominences within the illocutionary units (Comment) from those characterizing Topic units. Patients show a flattening of the Comment-prominence, reflecting impairments in performing the illocutionary activity. Reduced values of spectral emphasis and intensity variation also suggest a lack of engagement in communication. Conversely, Topic-prominence shows higher values for f0 standard deviation and spectral emphasis, suggesting effort when defining the domain of relevance of the illocutionary force. When comparing Topic and Comment-prominences of patients, the former consistently exhibit higher values across all parameters. In contrast, the non-pathological group displays the opposite pattern.

## 1. Introduction

Language and communication dysfunction characterize all the symptoms of schizophrenia. Verbal communication impairments appear among the symptoms as positive/negative thought disorder (Liddle et al., 2002; Kuperberg, 2010; DSM, 2013). The literature widely describes patients’ “thought disorders,” including poverty of speech, disorganization in the discourse, which is hard to follow, derailment and tangentiality with a loosening of associations (Bleuler, 1950; Andreasen, 1986; DSM, 2013). The impairments lead to difficulties in interpersonal communication for patients (Elvevåg et al., 2010) and damage pragmatic abilities, contributing to social dysfunction (Bowie and Harvey, 2008); moreover, it is possible to underline correlations between types of schizophrenic pathology and linguistic functioning (Bambini et al., 2022), the damage of which is associated with a reduced brain specialization (Cavelti et al., 2018; Boer et al., 2020). These phenomena depict an overall monotony in schizophrenic speech (Dovetto et al., 2015; Cresti and Moneglia, 2017).

To assess the psychopathology of schizophrenia, numerous evaluation scales have been employed since the 1960s.^1^ However, it has become evident that these scales rely on human judgment, necessitating fresh approaches or analyses to interpret the symptomatic heterogeneity of the disease accurately (Bambini et al., 2022), characterized by variations from one individual to another and within the same individual at different disease stages.

The present research focuses on the qualitative evaluation of linguistic profiles within schizophrenia. It deals with prosodic and pragmatic features that characterize speech productions in spontaneous interactions and takes a corpus-based approach. We will search for markers of schizophrenic speech at three levels of the prosody/pragmatic interface, which in principle may be responsible for the monotony effect: (a) the informational complexity of the utterance; (b) the disfluencies of the speech flow; and (c) the prosodic prominence of the information units.

The research exploits an existing dataset of spontaneous speech of a small number of patients (4 schizophrenic subjects) compared with a control group (23 speakers), which is not sex-aged matched. The validity of the quantitative difference between the number of schizophrenic patients (*n* = 4) and the control group (*n* = 23) lies in the corpus-linguistics method. For qualitative analyses, the comparison group is restricted to 4 speakers to guarantee the relevance of the comparison. The analysis should be considered as a preliminary proof of concept study.

The Language into Act Theory (L-AcT) is the theoretical framework adopted for the research. L-AcT focuses on the pragmatic role played by prosody in speech organization and is specifically designed for spontaneous speech corpora analysis (Cresti, 2000; Cresti and Moneglia, 2018). The framework provides explicit methods for speech segmentation into utterances (Moneglia, 2005) and for the annotation of information structure that are based on the hypothesis of a systematic correspondence between prosodic units and information functions (Cresti, 2000; Moneglia and Raso, 2014). L-AcT has been extensively applied to spoken Romance languages and tested on English, Japanese, and Chinese (Cresti and Moneglia, 2018; Cresti et al., forthcoming). Among the main achievements, the C-ORAL-ROM – C-ORAL-BRASIL collections of comparable spoken romance corpora (Italian, French Spanish; European Portuguese; Basilian Portuguese (Cresti and Moneglia, 2005; Raso and Mello, 2013), the DBIPIC crosslinguistic Information structure Data Base (Panunzi and Gregori, 2012), which allows comparative studies of speech organization in Italian, Spanish, English, and Brazilian Portuguese, and a Corpus-based Taxonomy of Illocution Acts based on the prosodic performance (Cresti, 2020). Praat (Boersma and Weenink, 2021) and Winpitch (Martin, 2004) voice analysis software are the analysis tools.

L-AcT has already generated studies focusing on schizophrenia in Italian and Brazilian Portuguese. It has been made the hypothesis that patients have a specific difficulty in building up utterances presenting a Topic (Rocha et al., 2022, forthcoming) while they show an atypical preference for post-nuclear units (Appendix; Dovetto et al., 2015; Cresti and Moneglia, 2018). This difficulty seems to emerge in complex discourse contexts where patients do less structured speech productions, with a statistically significant decrease in Topic and a relevant increase in Appendix (Costa, 2022). In addition, for what concerns Italian, it has been highlighted that schizophrenic speech records an abnormal quantity of pauses and retracing phenomena (Saccone and Trillocco, 2022), and that pauses characterize schizophrenic speech, specifically in turn-taking position (in line with Lucarini et al., 2022).

The paper is organized as follows. In 3.1, the complexity in schizophrenic speech is studied compared to controls by observing the amount of information in the utterance in terms of its length (MLU) and from the point of view of its informational complexity. Results, which only partially fit the expectations, give a measure of the atypical profile of schizophrenic speech considering the individual variability of patients. Values scored by patients will be compared to the controls and the general measures available for Italian (Cresti, 2005, p. 227; Saccone, 2022).

In 3.2, based on the segmentation of the speech flow into utterances and information units, a fine-grained analysis of disfluencies will be presented. Disfluencies, which strongly characterize schizophrenic speech, refer to hesitation phenomena and indicate the speaker’s effort in planning, production, and post-articulatory evaluation (Ginzburg et al., 2014). Disfluencies are dysfunctional (Allwood, 2017), “disturb” the flow of communication (Eklund, 2004), and are also pervasive in everyday language performance (Cresti, 2000). Pauses and retracing phenomena have been investigated face to their possible positions inside the turn and considering their qualitative characteristics.

Finally, in 3.3 prosodic analysis of pathological speech has been carried out, in line with the most recent research (Dickey et al., 2012; Compton et al., 2018; Lucarini et al., 2020, 2021, 2022). The focus is on prosodic prominences, a perceptual phenomenon that emphasizes linguistic segments compared to the surrounding context (Gagliardi et al., 2012; Lombardi Vallauri, 2014; Barbosa, 2019). Prominence is determined by a complex interaction of prosodic and phonetic/acoustic parameters, essentially pitch and force accents. Pitch accent refers to fundamental frequency values, while force accent refers to intensity and duration.

The relevance of the prosodic prominence parameter in schizophrenia is highlighted in Martínez-Sánchez et al. (2015): at the nucleus’ syllabic level, slowness in the movement of the f0 and different realization of risings (peaks) and fallings (valleys) emerge with lower values in patients. In particular, the greater the number of years since diagnosis, the lower the intrasyllabic trajectories of f0, and the greater the amount of time since the last relapse, the less intrasyllabic trajectories of f0.

Further studies underline a direct correlation between a lowering of f0 and negative symptoms of schizophrenia (see aprosody in Compton et al., 2018) as well as different pathologies such as depression (Silva et al., 2021), mutational falsetto, laryngeal carcinoma, and vocal cord polyps (Li et al., 2021).

Following the L-AcT approach, we will analyze acoustic indices specifically in the nucleus of the illocutionary unit of Comment and in the nucleus of the Topic Information Units whose prosodic profile presents prominence. To this end, we used the automatic script of Barbosa et al. (2019), which provides parameters to measure the movements of f0 and its variation. Spectral emphasis and intensity variation have also been calculated, correlating with a lack of engagement in communicative events (cf. Pellet-Rostaing et al., 2023).

The paper aims to highlight distinctive properties of the speech flow in patients with schizophrenia through empirical research and data retrieved specifically from spontaneous speech corpora. Spontaneous spoken language is the field of communication in which idea processing needs to be synchronized with the interaction; thus, observing patients’ speech in a spontaneous interactive environment enables us to examine the actual context in which the linguistic outcomes of the pathology manifest.

## 2. Materials and methods

### 2.1. Data collections

The research relies on a case study of schizophrenic speech recorded during psychiatric interviews (Corpus of Italian Spoken Pathological/Schizophrenic CIPPS, Dovetto and Gemelli,^2^ 2013; Dovetto et al., 2021), which has been intensively analyzed from the perspective of pragmatic and acoustic studies (Cresti and Moneglia, 2017; Saccone and Trillocco, 2022; Cresti et al., forthcoming) in comparison with a control-group of non-pathological spontaneous speech derived from the LABLITA corpus of spoken Italian^3^ (Cresti et al., forthcoming).

CIPPS collects about 9 h of recordings (44.270 tokens; 6.707 utterances) of 4 male speakers with Schizophrenia aged 35–45. Patients originate from Naples and metropolitan areas and are conventionally identified as A, B, C, and D.

The recording sessions are in the form of medical interviews between each patient and the psychiatrist and mainly consist of monologic excerpts due to the low presence of the doctor’s turns. The interviews are about daily habits or topics the patient wants to discuss. They have been originally manually transcribed with orthographic criteria based on Savy (2005). Transcripts have been adapted to the CHAT-LABLITA format (Moneglia and Cresti, 1997; MacWhinney, 2000, 2012), comprehending prosodic and pragmatic annotations.

The four patients differ in the severity of the pathology and are characterized by different subtypes of schizophrenia (no longer considered in the DSM5), reflected in the speech flow.^4^

The clinical characterization of the patients in CIPPS follows the approach of phenomenological psychiatry,^5^ which was strongly influenced by Husserl’s philosophy (Jaspers, 1963) and Heidegger’s existentialism (Binswanger, 1942). This perspective considers that, in the realm of the human, the explanation of behavior through the observation of regularity and patterns (*Erklärende Psychologie*) must be supplemented by an understanding of the “meaning-relations” experienced by human beings (*Verstehende Psychologie*). Patients’ experience is accessed through the clinician’s ability to “identify” with his psychic states (Jaspers). The clinical interviews collected in CIPPS are part of this attempt and are characterized by the maximum possible spontaneity and empathy.

In short, the diagnoses joint to the original data collection are as follows:

- Pre-delusional condition of *Wahnstimmung* without hallucinations.
- Paranoid schizophrenia with unstructured delirium without hallucinations.
- Paranoid schizophrenia with structured delirium and hallucinations.
- Paranoid schizophrenia with delirium.

Table 1 gives a summary of the corpus.

**Table 1:** Summary of CIPPS data.

Patient	Recording duration	Stretch of speech	Tokens	Utterances
A	2 h 30 m	1 h 3 m	2,563	619
B	3 h 58 m	3 h 43 m	30,021	4,204
C	2 h 8 m	1 h 26 m	10,409	1,552
D	28 m	17 m	1,277	332
Tot.	9 h 4 m	6 h 29 m	44,270	6,707

The context of the clinical interview of CIPPS is not replicable in a non-pathological population. For instance, the therapeutic goal influences the relationship; the doctor tries not to interrupt the patient and stimulates his language activities. The control group corpus (CORCON) collects 3 h and 57 min of spontaneous speech of 23 healthy controls recorded during interviews in a friendly and motivating environment on various subjects, such as the speaker’s life, work, habits, and family. For each recording, the interviewer is a friend or a well-known person by the main speaker. Most speakers are from Central Italy. Since this control group is not balanced in terms of age, gender, diatopic, diaphasic, and diastratic characteristics (see Table 2), two subsets have been selected for specific analyses (SAMP and SAMP(100)). The main control group only compares the mean length of terminated sequences (MLU) and silences within the speech flow.

**Table 2:** Summary of groups’ demographic data.

	Gender	Age	Geographic origin
CIPPS	4 men	35–45	Naples
CORCON	17 men, 6 women	26–40 (6), 41–60 (10), >60 (7)	Florence (15), Siena (3), Arezzo (2), Milano (1), L’Aquila (1), Terni (1)
SAMP	4 men	35–45	Florence

SAMP was used for fine-grained analyses, such as information structure and the retracing phenomena, for which we need a more precise comparison selection concerning gender, age, and qualitative features of the interaction. To reduce the differences with the communicative context of CIPPS, SAMP selects four interviews, three about the work experience in life and one on the psychological problems experienced in family life, thus maintaining the presence of a main speaker and a solid motivation to interact in the intersubjective relation.^6^

SAMP(100) is a balanced subset of SAMP consisting of each speaker’s first 100 terminated sequences; it was used for fine-grained acoustic research on prosodic prominence.

Table 3 gives a summary of CORCON and the two subsets.

**Table 3:** Summary of control groups speech data.

Control group	Speakers	Stretch of speech	Tokens	Utterances
CORCON	23	3 h 57 m	34,398	4,016
SAMP	4	1 h 8 m	9,108	966
SAMP(100)	4	33 m	4,639	436

### 2.2. Methods and theoretical framework

The research is carried out within the Language into Act Theory (L-AcT, Cresti, 2000; Moneglia and Raso, 2014; Cresti and Moneglia, 2018). According to L-AcT, the utterance is the primary referring unit for the analysis of spoken language, which results from pragmatic activities by the speaker; it is autonomous and conveys an illocutionary act. The segmentation of the speech flow into utterances is achieved through perceptual judgments into terminated sequences (TS) identified through their prosodic profile (Izre'el et al., 2020). Subsequently, TSs are segmented into prosodic/information units, showing their information structure independently from their syntactic form. Thus, prosodic boundaries recognized in the speech flow provide its segmentation into utterances (terminal prosodic boundary, ‘//’) and smaller chunks, i.e., prosodic-information units (non-terminal prosodic boundary, ‘/’).

Through prosody, it is also possible to define which unit inside the utterance bears the illocution and, therefore, carries the pragmatic and prosodic autonomy of the sequence; this unit is named Comment (COM) and is necessary and sufficient to form an utterance. The prosodic contour of the COM can be described as a *root* unit (‘t Hart et al., 1990); it widely varies as a function of its illocutionary value.

According to L-AcT, utterances can be simple or complex regarding their information structure: a simple utterance consists of only one prosodic/information unit, necessarily a COM bearing an illocutionary value (see example 1); conversely, a complex utterance consists of more than one prosodic/information unit, one of which is always the COM (see example 2, in which the COM is underlined).

1. faccio un po’ di tutto // [LABLITA: prvmnl01-cami]

I do a bit of everything//

1. e poi/niente// [LABLITA: prvmnl01-cami]

and then/nothing//

When an utterance is complex, the COM is supported by other units. Therefore, apart from the units that bear the illocution, for our goals, it is relevant to introduce two units identified within the L-AcT theoretical framework: Topic and Appendix.

Following Moneglia and Raso (2014), the Topic (TOP) provides the field of application for the illocutionary force of the Comment; it supplies the semantic representation of the domain of facts to which the illocutionary act refers (“pragmatic aboutness”). That is, utterances without a TOP necessarily refer to the context. Regarding its distribution, TOP units always precede the COM and have a *prefix* prosodic contour (‘t Hart et al., 1990; Cavalcante, 2016). On the other hand, the Appendix (APC) integrates the text of the COM and necessarily follows it. APC is performed with a *suffix* prosodic contour (in ‘t Hart’s terms) and does not have functional prosodic prominence (Cresti et al., forthcoming).

Identifying these units leans on recognizing and perceiving relevant prosodic movements – *root* for COM; *prefix* for TOP; *suffix* for APC. Both *prefix* and *root* prosodic contours can comprise a preparation and a nucleus. The nucleus corresponds to the minimal prosodic contour sufficient to perform the information unit; its contour can be composed of a simple movement (rising/falling/holding) or several movements aligned to the syllables participating in the contour (Cresti and Moneglia, 2023); thus it is possible to identify a prosodically prominent part in both units of Topic and Comment whose relevance is connected to their functional value.

**Figure 1:**
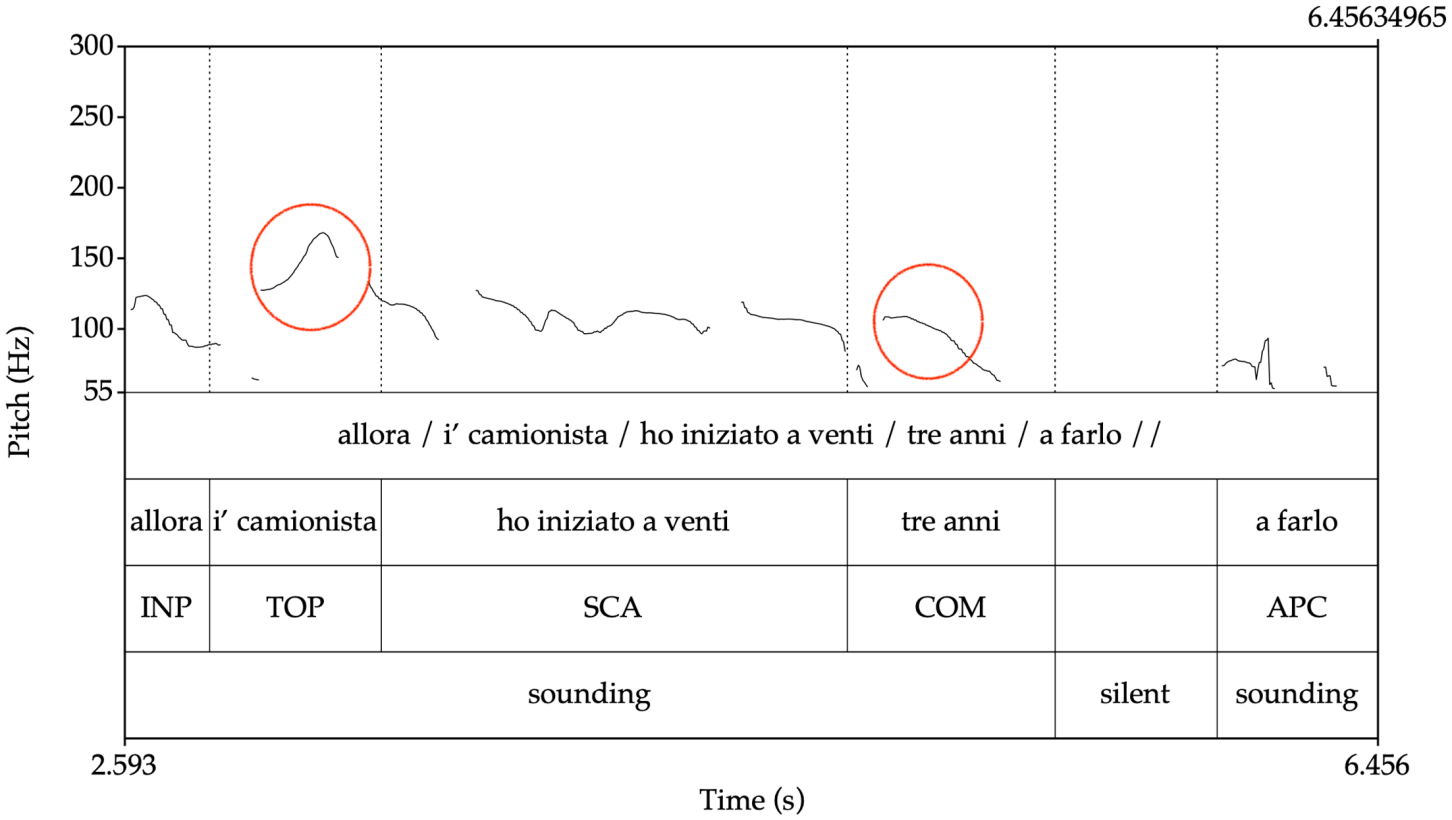
Annotation of a complex utterance.

See in (3) an example of a complex utterance with the information structure of TOP/COM/APC; Figure 1 shows the prosodic contour and the text labeled following the information tags.

1. allora / i’ camionista /^TOP^ ho iniziato a venti / tre anni /^COM^ a farlo //^APC^ [LABLITA: prvmnl01-cami]

so/ the trucker/I started at twenty/three/doing it//

In Figure 1, the prominences of TOP and COM are circled in red. They include the rising movement, the peak, and the falling movement.

The previous examples (1), (2), and (3) show utterances in which only one unit bears the illocutionary force (‘faccio un po’ di tutto’ in 1; ‘niente’ in 2; ‘ho iniziato a venti / tre anni’ in 3); however, empirical studies in spontaneous speech, in particular in monologs, led to the identification of a different kind of terminated sequences in which more than one unit bears an illocution. It is usually the case of long excerpts of speech flow, in which the speaker develops a thought through a chain of semantic *foci*, and the illocution tends to remain unchanged (usually assertive). See an example in (4):

1. l’ ho fatto per diversi anni / poi mi sono messo in proprio / s’ è creato una piccola azienda / da una piccola azienda viene poi / quell’ altra / e via // [LABLITA: prvmnl01-cami]

I did it for several years /then I branched out on my own /we set up a small company/from a small company then comes/ another/and so on//

Each unit in (4) bears a weak illocution. This type of TS, named *stanza*, has specific characteristics such as a monotonous prosodic trend and a “step-by-step” adjunctive structure. They are usually present where the implementation of speech is less interactive, as in monologs, and the speaker focuses on the semantic elaboration of the text (Cresti, 2005; Panunzi and Scarano, 2009; Saccone, 2022). Inside a stanza, the units bearing an illocutionary value are named Bound Comments (COB) since they are linked together (bound) through prosodic and pragmatic features.

Assuming the L-AcT framework, automatic temporal and acoustic measurements of the signal are linked to the perceptual processing of linguistic data. The sound is aligned with the transcription and segmented both at the utterance level and, more specifically, at the information unit level.

Based on this multilayer annotation process, the analysis will explore (i) the structure and length of the utterance; (ii) speech disfluencies such as pauses and retracing phenomena (false starts, repetitions, corrections); (iii) a chosen set of acoustic parameters that highlight perceptual prosodic correlates of the schizophrenic atypia (mainly based on f0 and intensity).

On the first point, according to L-AcT, the audio files are segmented into TS (utterances and stanzas), and subsequently in information units. The segmentation in TS allows the quantitative measurement of their length in word numbers, while the segmentation in information units allows the qualitative measure of the information strategies adopted by each speaker.

Regarding pauses, as already stated in Andreasen (1986) and cf. Liddle et al. (2002), one of the symptoms of schizophrenia is *blocking*, i.e., the interruption of thought followed by a phase of silence that can last from a few seconds to a few minutes. In Goldman-Eisler (1961) and Banfi (1999), the length of the pauses is a clear distinction between pathological and non-pathological speech, and in Cannizzaro et al. (2005) the abnormal quantity of silence is highlighted as a clear marker of patients’ speech. The most recent linguistic studies, albeit with different approaches, confirm these results (Heldner and Edlund, 2010; Fors, 2011; Dodane and Hirsch, 2018; Bambini et al., 2022). Lucarini et al. (2021) do a conversation analysis of schizophrenic speech and observe a specific correlation between pause duration and negative symptoms.^7^

CIPPS and CORCON audio files are segmented into “sounding” and “silent” based on Praat’s script. All silences over 150 ms are considered and grouped quantitatively by duration thresholds and qualitatively by their position. Exploiting the L-AcT approach, position labeling distinguishes pauses between utterances of the same turn and between information units within the utterance. Moreover, considering the latest generation typological approach (cf. *inter-tours* and *intra-tours* in Dodane and Hirsch, 2018; *gaps*/*lapses* and *pauses* in Heldner and Edlund, 2010; Fors, 2011), each silent is labeled according to the following types:

- T (<turns): When the pause occurs between the turns of the two different speakers, it is, in principle, an index of the interviewed responsiveness in the intersubjective interaction. Therefore, the count of pauses T is limited only to pauses “before” the turn because they are an index of the patient’s reaction time to the interlocutor’s questions^8^
- UT (<utterances): When the pause occurs between two utterances of the same turn by the same speaker, it refers in principle to the difficulty of maintaining the turn programming a new speech act.
- IU (<informational units): When the pause occurs between two information units of the same utterance, it deals with the problems in conceiving the locutionary content of the information unit.

One added value of the CHAT/LABLITA transcription is the annotation of retracing phenomena such as hesitations, repeated words or fragments of words, false starts, and repairs. Often considered an *error* (Hieke, 1981) or, more generally, an *alteration* (Ginzburg et al., 2014), retracing is a fragmentation of the locutionary program, which is widely present in spontaneous speech performance (Cresti, 2000). In our transcription format, the symbols * and [/] respectively mark a retracted unit’s beginning and end. The system allows accuracy in identifying the retracing events and the number of retracted tokens. Data were analyzed based on the different positions in the terminated sequences (at the very beginning of a TS -Start of TS-; inside a TS -Inside TS-; and at the beginning of an information unit -Start of IU-inside TS), distinguishing between isolated episodes and successions of retracing, called *chains*.

Lastly, to highlight perceptual prosodic correlates of the schizophrenic atypia, prominences are manually identified on Praat for each COM- and TOP unit. Four acoustic parameters are selected for each prominence: (i) f0 mean, the mean of the average number of oscillations of the vocal folds per second, starting parameters for the voice description; (ii) f0 standard deviation, which measures the variability of the f0 (connected to the neuromuscular control and the regularity of laryngeal vibration of the vocal folds in Lopes et al., 2017); (iii) Spectral emphasis, which measures the vocal effort (Traunmüller and Eriksson, 2000) and correlates with the energy expended during the speech flow; and (vi) the coefficient of intensity variation, which reports the ratio between the mean and the intensity standard deviation.

## 3. Results

### 3.1. The structure of the utterance

The direct relation between prosody and pragmatics foreseen by the L-AcT theoretical framework allows for outlining a first sketch of the linguistic complexity and productivity in the 4 patients compared to the control groups based on the annotation of the terminated sequences and their division into prosodic units. We will first observe the measurements for the Mean Length of Utterance (MLU); subsequently, we will report data about the inner structure of the terminated sequences (information structure).

#### 3.1.1. Mean length of utterance

**Figure 2:**
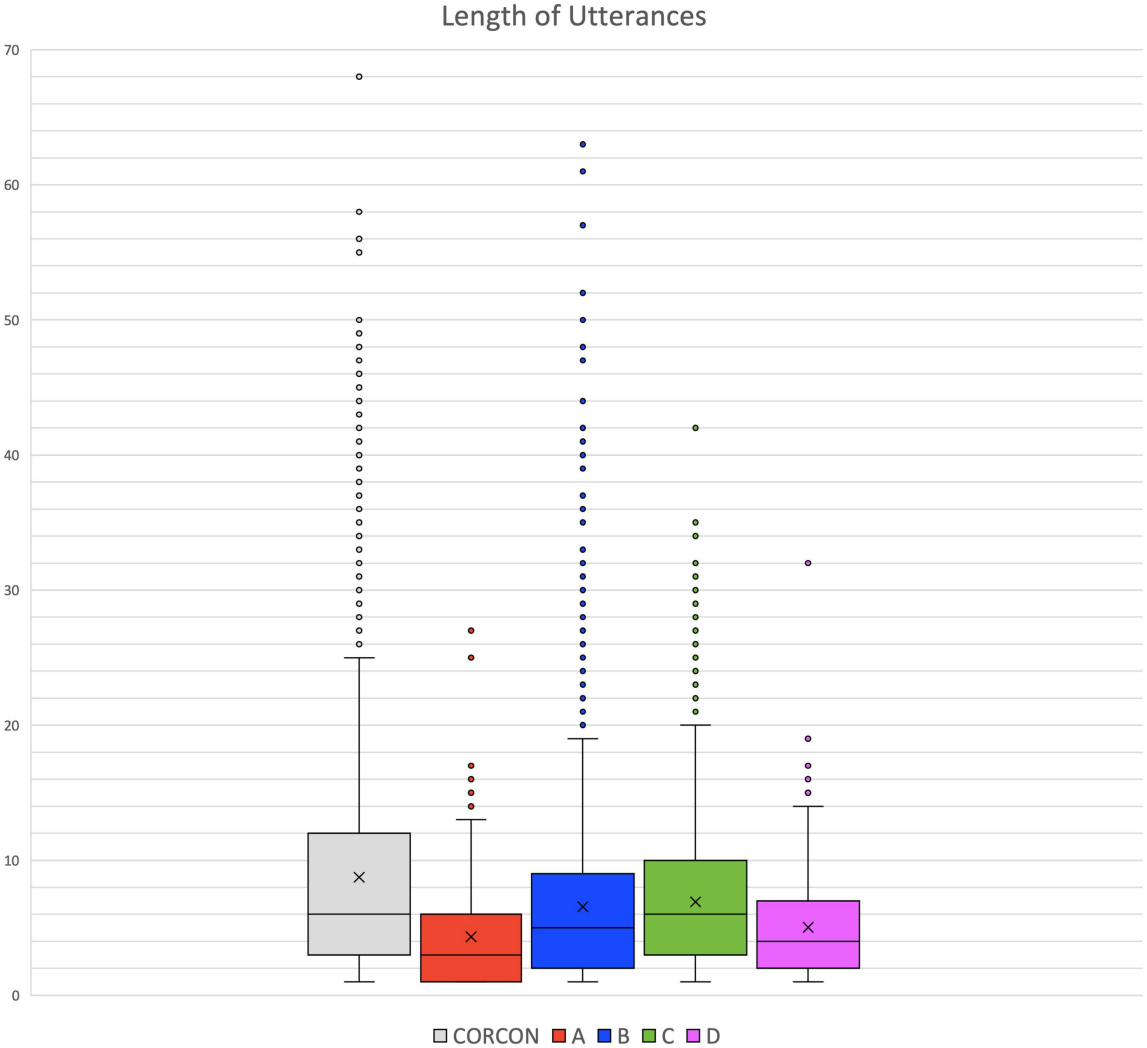
Length of utterances.

The MLU reflects the complexity of the spoken structures in terms of the number of words contributing to the semantic content of a TS.^9^ The analysis has been carried out on the whole set of corpora under consideration (CIPPS and CORCON). Figure 2 and Table 4 show the measurements of length for each utterances, the mean values per patient (colored box plots), and the collected measurements for the control group (distribution in the gray box plot).

**Table 4:** MLU values.

	MLU
A	4.3
B	6.5
C	6.9
D	5
CORCON	mean per speaker: 5.2–15.7 (average value: 8.8)

The 4 boxes of CIPPS extend behind the CORCON mean (indicated with an ‘x’ inside the gray box), and when considering whiskers, the CIPPS extension never exceeds that of CORCON. Patients B (blue box) and C (green box) show, on average, closer proximity to the controls (B: 6.5; C: 6.9; CORCON: 8.8 words/utterance), while A (red box) and D (pink box) exhibit lower MLU values (A: 4.3; D: 5). For patients A and D, more than a quarter of their utterances consist of a single word, whereas this applies to only 1 out of 20 of the CORCON’s utterances. The high peaks in the control group variation (mean maximum rate: 15.7 words/utterance) correlate with the monologic context of the recordings.^10^ Schizophrenic speech is characterized by qualitatively shorter utterances, where the discourse is structured in smaller chunks.

To assess statistical significance, the Kruskal-Wallis test for not normally distributed data has been conducted, but it did not yield *p*-values <0.05 (A: *p*-value = 0.1409; B: *p*-value = 0.578; C: *p*-value = 0.2688; D: *p*-value = 0.1029).

#### 3.1.2. Information structure and complexity

Further analysis has been performed to examine how TSs are structured and evaluate their complexity, considering whether TSs give rise to utterances or stanzas and whether utterances consist of a single COM unit or are structured from an informational point of view. The analysis has been processed on a CIPPS Sample of 4,892 tokens (755 terminated sequences); the chosen excerpts are the first 15 min of each patient.^11^ TSs have been segmented into units and labeled following their prosodic form and information function; hence, data concerning the information structure were extracted.^12^ Schizophrenic data are compared with the control group SAMP. Table 5 presents the comparison. For these parameters, applying a statistical significance test was impossible as the initial samples were not calibrated for statistical comparison.

**Table 5:** Information structure: classification of terminated sequences.

	Terminated sequences	Simple utterances	Complex utterances	Stanzas
A	77	39 (50.6%)	32 (41.6%)	6 (7.8%)
B	274	125 (45.6%)	109 (39.8%)	40 (14.6%)
C	202	86 (42.6%)	84 (41.6%)	32 (15.8%)
D	202	93 (46.0%)	84 (41.6%)	25 (12.4%)
CIPPS	755	343 (45.4%)	309 (40.9%)	103 (13.7%)
SAMP	966	304 (14.2–42.6%; mean: 31.4%)	448 (38.7–58.2%; mean: 46.4%)	214 (15.6–35.0%; mean 22.2%)

Regarding the frequency of simple utterances, the average value for the control group in SAMP (31.4%) aligns with the trend of Italian monologic informal speech observed in previous studies (Cresti, 2005, p. 227), i.e., 30.5%. However, the variation among the four speakers is high (14.2–42.6%); two speakers produce nearly 15% of simple utterances, while the others are close to 43%. Despite individual differences, complex TSs (complex utterances and stanzas) overtake simple ones in non-pathological speech. In contrast, the trend of schizophrenic patients is less heterogeneous and shows a reduced gap between simple and complex TSs. CIPPS simple utterances always outnumber the control percentage (≥42.6%): For patient A, simple utterances go slightly beyond half of the total (50.6% simple), while in the other three (B, C, and D), the percentage of complex TSs increases moving closer to the non-pathological distribution.

Beyond the relation between simple utterances and complex TSs, Table 5 shows the frequency of stanzas. As pointed out in the method section, a high presence of stanzas is expected in monologs. Previous corpus-based studies (Saccone, 2022) reveal that in Italian speech, the number of stanzas increases from 6.3% of TSs in dialogs/conversations to 19.8% in monologs.^13^ The recurrence of stanzas allows the speaker to extend his turn, performing his thought chunk by chunk, using small pieces of information, each with a weak illocutionary value. Using these macrostructures requires the speaker to have an overall idea of what should be said, even if the content can be progressively planned during the production of the discourse. Given these premises, we might expect a low presence of stanzas in schizophrenic speech where thoughts are, in principle, less organized. Again, the variation of the percentage of stanzas among the four controls is high (15.6–35.0%) with an average of 22.2% of TSs. CIPPS’ rates are approximately under the minimum of the controls (15.6%), and, again, the value decreases to 7.8% for patient A.

Data, therefore, indicate a tendency of CIPPS patients to reduce the informational complexity of the speech flow, both about the information structure of the utterance (as expected in Dovetto et al., 2015; Cresti and Moneglia, 2018) and also about the stanzas.

#### 3.1.3. Information units

Lastly, the inner composition of TS (complex utterances and stanzas) has been analyzed by looking at the frequency of Topic (TOP) and Appendix (APC) units. Data show relevant intersubjective variation for non-pathological and schizophrenic speech, as summarized in Table 6. The reported values indicate the percentages of TSs with TOP/APC. Also, applying a statistical significance test was impossible for these parameters as the initial samples were not calibrated for statistical comparison.

**Table 6:** Information structure: presence of topic and appendix.

	Terminated sequences	TOP	APC
A	77	6 (7.8%)	6 (7.8%)
B	274	71 (25.9%)	17 (6.2%)
C	202	36 (17.8%)	15 (7.4%)
D	202	24 (12.4%)	22 (10.9%)
CIPPS	786	137 (17.4%)	60 (7.6%)
SAMP	966	315 (4.9–64.1%; mean: 32.6%)	57 (3.7–8.8%; mean: 5.9%)

Regarding TOP, both groups show a variable behavior, especially the control one. CIPPS values are always beyond the control’s mean (<32.6); while staying in the lower part of the distribution, they are still included in the range of variation of SAMP. On the other hand, the presence of APC shows the opposite trend: all four patients’ values are distributed above the controls’ mean (>5.9%), and in one case (D), APC frequency overcomes the controls’ maximum (10.9%).

The TOP is more frequent than the APC in every speaker (except A, which shows the same number of both). Still, the CIPPS trend is remarkably different from the non-pathological ones since the percentages for the two units in schizophrenic patients are much closer, which leads to a higher relative frequency of APC. Indeed, the reported number of APCs is noteworthy, showing a marked preference for delocalizing and defocusing information in the right periphery of the utterance.

### 3.2. Disfluencies

Based on the segmentation of the speech flow into TSs and information units, a fine-grained analysis of disfluencies is presented here, focusing on pauses and retracing phenomena, which have been investigated for their distribution inside the turn and their qualitative characteristics.^14^

#### 3.2.1. Pauses

**Figure 3:**
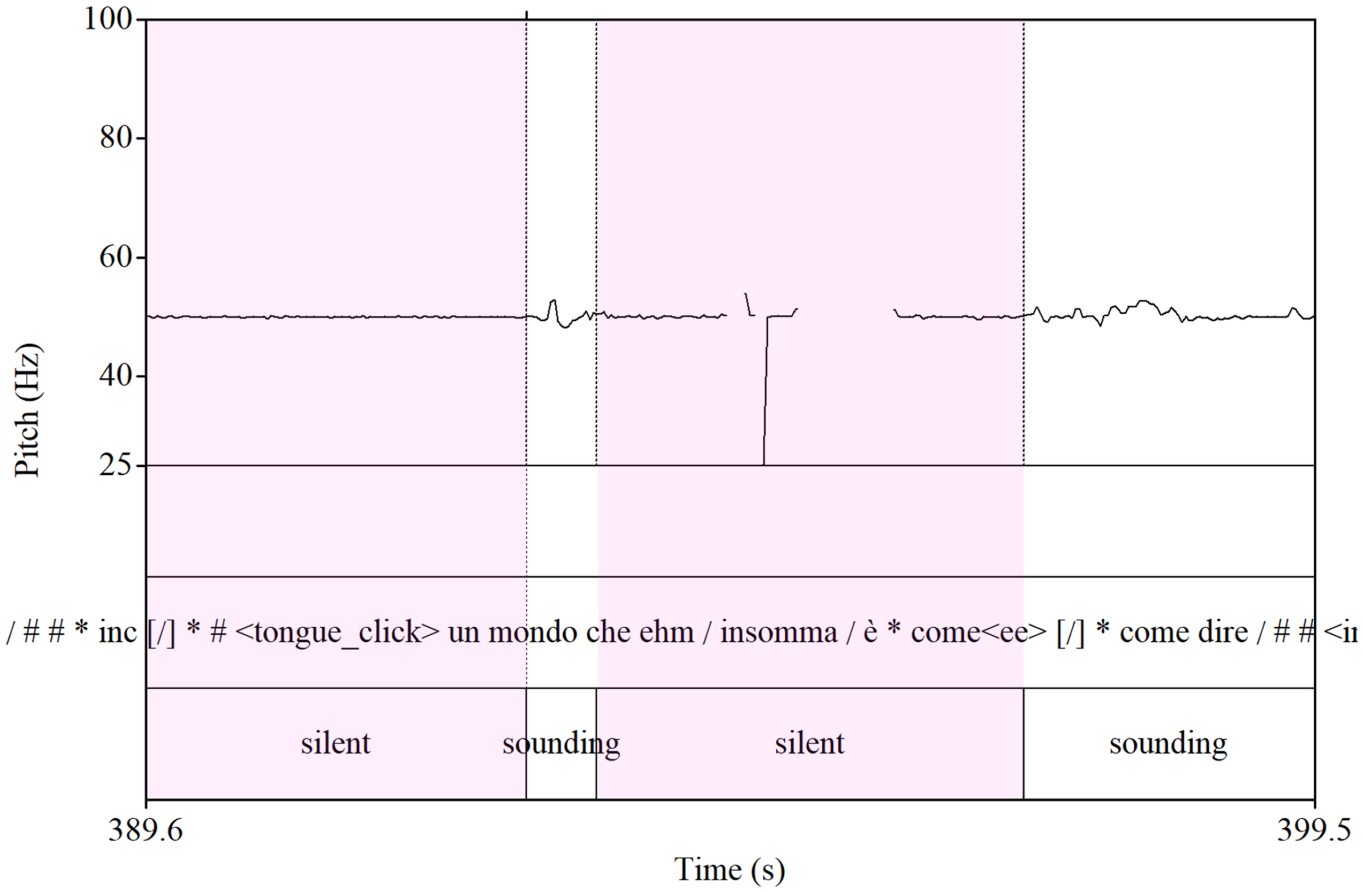
Silent and sounding in schizophrenic speech.

For the analysis of the pauses, the Control Group is CORCON. Pauses have been automatically identified in the signal and manually classified in terms of inside/between utterances and turn-taking pauses and length (for a detailed description of the data processing, see Saccone and Trillocco, 2022; Trillocco, forthcoming). Related to the automatic identification, the sounding/silent script on Praat was used and manually checked by two revisors.^15^ In Figure 3, an excerpt from CIPPS (patient A) shows the abnormal length of pauses in schizophrenic speech: pink parts are pauses, and white parts are speech.

Silences so identified have been studied considering their position and duration.

**Figure 4:**
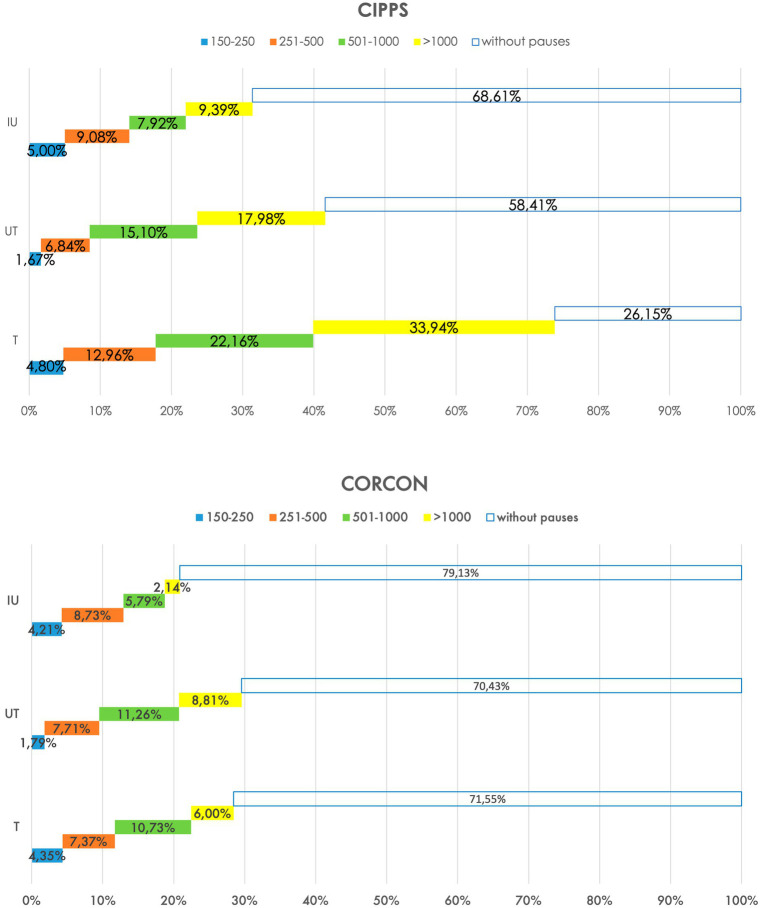
Duration of pauses.

**Figure 5:**
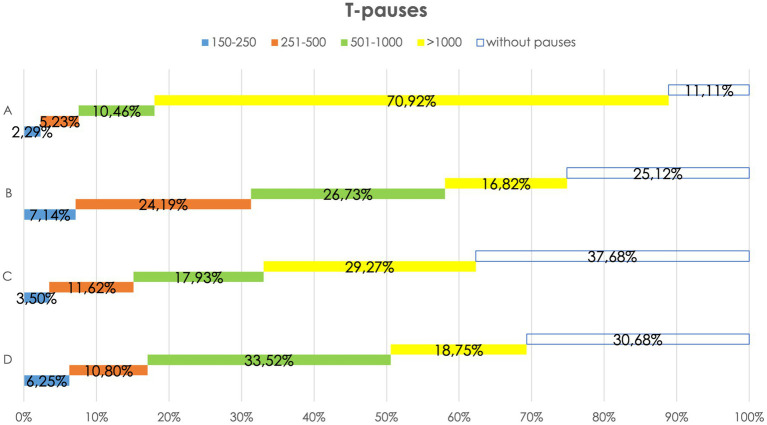
T-pauses in CIPPS.

The minimum threshold established (150 ms) corresponds to the average duration of the stop consonants.^16^ According to the literature (Duez, 1985; Dovetto and Gemelli, 2013), only four duration thresholds have been considered: 150–250 ms, 251–500 ms, 501–1,000 ms, and > 1,001 ms. The percentages of pauses of each type were then calculated in relation to their position. Figures 4, 5 present the results.

Firstly, we observe in Figure 4 the comparison between CIPPS and CORCON: the length of transparent bars is shorter in the CIPPS for T pauses (26.15% vs. 71.55%) and UT pauses (58.41% vs. 70.43%), revealing the greater pervasiveness of silences in relation to turn-taking and between utterances. At the IU level, the two groups show a lower difference (68.61% vs. 79.13% without pauses).

The difference of the yellow bars (pauses >1 s) is the most evident data: their length is more extensive for the CIPPS regardless of the type of pause considered (IU: 9.39% vs. 1.40%; UT: 17.98% vs. 8.81%; T: 33.94% vs. 6.00%). In controls, these only sporadically exceed 2 s; in the pathological, they can even exceed the 20s.

Moreover, the same trend is observed for the green bars UT and T (500–1,000 ms), longer than those of non-pathological speech (UT: 15.10% vs. 11.26; T: 22.16% vs. 10.73%), in line with expectations (Banfi, 1999; Heldner and Edlund, 2010).

The trend is markedly different regarding T pauses: while in most cases, there is no pause at the start of the turn in the non-pathological (71.55%), in the pathological, only 26.15% of turns do not present silences before. This difference does not regard short pauses (almost 5% in both corpora) but mainly pauses longer than 500 ms. In short, pauses do not characterize locutionary programming but mainly occur between utterances and in a marked manner at turn-taking.

Observing the various patients confirms the peculiarity of long pauses at the turn’s start. Figure 5 reports individual differences: Patient A very rarely (11.11%) starts a turn without silence, while B, C, and D slightly more often (25.12, 37.68, and 30.68%). The turn-taking delay, recently observed by Lucarini et al. (2022), is confirmed.

#### 3.2.2. Retracing phenomena

Retracing can be associated with both repetition (see examples 5, 6, and 7a) or modification (7b) of words; when the locutionary content is repeated, it can be total (5, 6) or partial (7a).

Retracing can occur in different positions of the terminated sequences: at the very beginning of a TS (Start of TS), otherwise inside a TS (Inside TS); the second case can be further split into two classes to isolate the retracing phenomena occurring inside a complex TS at the beginning of an information unit (Start of IU-inside TS). Retracing phenomena can occur in isolated episodes (5, 7a, 7b) or successions (6), called *chains*.

See the following examples:

1. Start of TS

*pe' [/] pe' dargli un colore più uniforme // [LABLITA: fammnl02-fale]

*to [/] *to give it a more uniform color*//

1. Start of IU-inside TS

i' ramo / *&d [/] *&d [/] d' un noce / gl' è più chiaro d' i' fusto // [LABLITA: fammnl02-fale]

the branch/ *of [/] *of [/] of a walnut/is lighter than the trunk//

1. Inside TS

- a casa mia *s’ era [/] gl’ eran poveri / e quindi ‘un c’ era / tanto da mangiare // [LABLITA: fammnl02-fale].

at home *we were [/] they were poor / and so there wasn’t / so much to eat //.

- ha fatto *le [/] il tecnico industriale // [LABLITA: pubdlr12-vefa].

he went to *the-PL-F [/] *the-SN-M technical industrial institute* //.

Once all the retracing phenomena of CIPPS and control groups had been labeled, data were analyzed to verify possible differences.

##### 3.2.2.1. Retracted tokens and units

**Figure 6:**
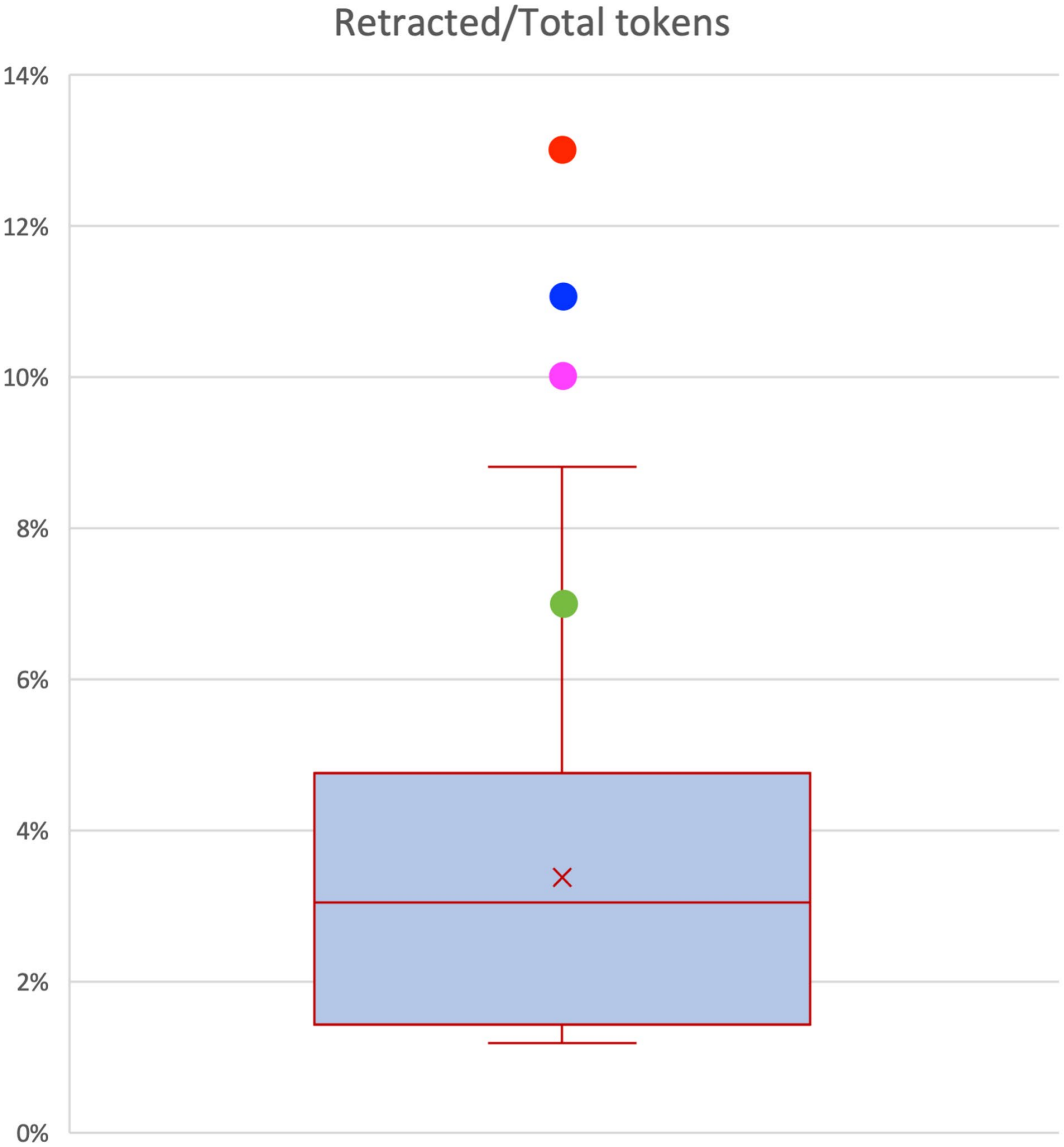
Retracted/total tokens.

We analyzed the phenomenon concerning the number of tokens produced (retracted tokens vs. total tokens) and the number of information units in which speech is articulated (retracing phenomena on information units) in both corpora.^17^ The results, summarized in Figure 6, show the tendency to produce retracing phenomena in schizophrenic speech.^18^

While the box represents the distribution of values in the control group, the colored dots indicate the 4 CIPPS patients, showing the incidence of the retracing phenomena on the number of tokens. All the patients (A: 13.19%; B: 11.44%; C: 6.72%; D: 10.02%) outnumber the mean distribution of the controls (1.43–4.76%).

##### 3.2.2.2. Single episodes and chains

A fine-grained analysis is carried out to display the quantity and typology (single episodes/chains) of retracing phenomena related to the different types of terminated sequences. CIPPS data refer to the extract of Table 6, while those for the control group refer to SAMP.

In Table 7, the frequency of single retracting episodes is reported. The values are calculated by dividing the number of single episodes by the number of not retracted information units, according to the different types of terminated sequences in which they appear:

**Table 7:** Single episodes and chains: CIPPS and control group.

		Terminated sequences	Simple utterances	Complex utterances	Stanzas
Single episodes	CIPPS	9.02%	7.63%	11.26%	14.53%
	SAMP	5.30%	3.38%	5.70%	5.47%
Chains	CIPPS	3.31%	2.93%	3.45%	3.33%
	SAMP	1.15%	0.82%	1.66%	0.95%

The difference between the two groups manifests in the frequency of single retracing episodes for TS, which almost doubled in CIPPS (9.02% vs. 5.30%).

The trend remains roughly the same in the different types of terminated sequences: the percentage of single retracing episodes is more than double in simple (7.63% vs. 3.38%) and complex utterances (11.26% vs. 5.70%), while the greatest atypia is found in stanzas (14.53% vs. 5.47%), which is the type of TS less frequent in schizophrenic speech (13.7% vs. 22.2%, see Table 5). This highlights the difficulty in CIPPS to produce a more complex structure (complex utterances and stanzas).

The difference between single episodes and chains concerns the “intensity” of the disfluency phenomenon: a retracing chain indicates greater difficulty processing a single information unit. Retracing chains generally appear to be a typical trait of stuttering but are also present in the non-pathological, albeit with very low percentages.^19^ Table 7 shows the frequency of retracing chains in the two corpora.

These data show that schizophrenic patients produce roughly three times more chains in simple utterances (2.93% vs. 0.82%), complex utterances (3.45% vs. 1.66%), and stanzas (3.33% vs. 0.95%). Therefore, this type of disfluency seems associated with the disease in a more substantial way than single episodes.

##### 3.2.2.3. Distribution

Regarding the distribution of retracing inside the terminated sequence, it might be relevant to observe if a speaker retracts the first words of a terminated sequence (Start of TS) or retracts words when the unit and the TS are ongoing (Start of IU-inside TS or Inside TS). The two cases seem to respond to different causes of the retracing; the first is most likely related to uncertainty in building the locutionary content in its connection to the illocutionary programming, while the second concerns the locutionary level only since the illocutionary activity has already been conceived and planned.

Table 8 summarizes the comparison between pathological and non-pathological speakers.^20^

**Table 8:** Distribution of retracing phenomena.

	Start of TS	Start of IU-inside TS	Inside TS
A	17.6%	41.0%	41.4%
B	10.6%	59.5%	29.9%
C	21.9%	35.5%	42.6%
D	16.1%	49.4%	34.5%
CIPPS	12.6%	54.9%	32.4%
CORCON	13.1%	46.1%	40.8%

In both corpora, retracing phenomena occur above all when the utterance is ongoing (Start of IU-inside TS and Inside TS), while the position Start of TS rarely holds retracted words (CIPPS: 12.6%; CORCON: 13.1%). This trend is emphasized in B, who reports the lowest percentage at the Start of TS (10.6%) and the highest uncertainty in the locutionary processing at the Start of IU, i.e., after the first information unit of a complex TS (59.5%). No specific pathological trend emerges from this study. According to this data set, retracing characterizes most as a disfluency at the locutionary level, and no particular influence by the pragmatic level can be noted in patients.

### 3.3. Prosodic prominence

To study the prosodic prominence as a possible marker of the pathology, we have independently analyzed acoustic indices in the illocutionary unit of Comment and in the unit of Topic.^21^

The nucleus of the *root* and *prefix* prosodic units, corresponding to the minimal prosodic contour sufficient to perform the information function, is perceptively identified and is selected as the prominence, as highlighted in Figure 1.

The perceptive choice is validated using the values of f0, intensity, and duration observable in the spectrogram. For the replicability of the procedure, the prominence is segmented on the speech wave following a specific workflow:

- The movement starts with a rise of the f0, reaches a peak, and ends with a fall.
- Since the prominence can often concern only portions of words, in order not to break the semantic unity, it is arbitrarily established to include up to a maximum of two syllables before and two after the entire movement considered.
- The segment thus identified is labeled with the number of syllables of which it is composed^22^;

The analyses are conducted on the first 100 TSs for each patient and control of SAMP(100) using an automatic script (Barbosa et al., 2019).^23^

The measured acoustic parameters for each prominence are f0 mean and f0 standard deviation^24^; spectral emphasis (*emph*) that measures the vocal effort (Traunmüller and Eriksson, 2000); intensity variation coefficient (*cvint*) that reports the ratio between the mean and the intensity standard deviation.

In this case as well, to assess statistical significance, the Kruskal-Wallis test for not normally distributed data has been conducted, but it did not yield significant results (find in the footnotes below the report of the values per each parameter).

Table 9 summarizes the results obtained for COM and TOP in the two corpora. Data are reported as a whole and for each speaker.

**Table 9:** Acoustic parameters of Comment and Topic prominences.

COM-prominences							
	n COM	f0mean Hz	f0mean st	f0sd Hz	f0sd st	emph	cvint
A	89	59.29	69.30	18.82	1.96	0.58	1.04
B	83	101.43	75.64	63.10	7.60	3.56	4.14
C	71	100.44	75.82	44.39	6.01	2.35	3.95
D	69	83.39	72.33	44.17	4.81	1.69	2.1
**CIPPS**	**312**	**85.2**	**73.14**	**42.02**	**5.01**	**2.02**	**2.78**
cami	56	110.34	80.75	35.19	3.99	2.11	7.84
fale	46	108.57	80.61	26.37	3.37	3.56	9.57
pell	62	135.65	84.31	19.39	2.31	2.91	4.28
vefa	76	140.28	84.37	31.16	3.18	4.52	6.13
**SAMP(100)**	**240**	**126.02**	**82.79**	**28.14**	**3.18**	**3.36**	**6.72**

TOP-prominences							
	n TOP	f0mean Hz	f0mean st	f0sd Hz	f0sd st	emph	cvint
A	7	107.43	73.29	37.67	3.23	1.76	1.8
B	23	142.48	82.39	66.80	6.99	4.83	3.38
C	16	155.38	84.88	53.67	6.81	3.86	4.44
D	3	109.33	79	33.25	5.69	2.93	2.33
**CIPPS**	**49**	**139.65**	**81.69**	**56.29**	**6.31**	**3.97**	**3.44**
cami	38	154.29	86.47	27.43	2.87	2.84	5.02
fale	29	117.52	82.45	13.10	1.60	3.38	6.59
pell	11	134.64	84.36	7.43	0.84	1.82	3
vefa	18	133.44	84.56	22.45	2.49	3.58	5.22
**SAMP (100)**	**96**	**137.02**	**84.66**	**19.77**	**2.17**	**3.03**	**5.32**

In bold, corpus average values.

#### 3.3.1. f0mean

Comparing *f0mean* in the two groups, we can observe that the values for Comment are lower in CIPPS (85.20 Hz and 73.14st) than in SAMP(100) (126.02 Hz and 82.79st). On the contrary, for the Topic, the values of the *f0mean* in CIPPS (139.65 Hz and 81.69st) are similar to those of the non-pathological group (137.02 Hz and 84.66 st).

In schizophrenic speech, there is a higher *f0mean* for TOP-prominences (81.69st) compared to the COM-prominences (73.141st). For the control group, the two values are roughly equivalent (82.79st for the COM-prominences and 84.65st for the TOP-prominences).^25^

#### 3.3.2. f0sd

In principle, *f0sd* in COM-prominences might correlate with the variability of illocutions, so the initial hypothesis is that pathological speech, which is perceived as monotonous, might show low values of *f0sd*.

Nevertheless, although the *f0mean* values are lower for schizophrenic speech, the COM-prominences have higher *f0sd* values in CIPPS (42.02 Hz and 5.01st) than in SAMP(100) (28.14 Hz and 3.18st). The higher *f0sd* in pathological speech is even more evident for the TOP-prominences (56.29 Hz and 6.31st), almost three times those of the control group (19.77 Hz and 2.17st).

Further observations rely on the different features of the two information units. While there is a great variety of illocutions, we only know three prosodic profiles for the Topic (Cavalcante, 2016); hence, a higher *f0sd* in the Comment might be expected. This hypothesis is confirmed by the data of non-pathological speech (COM-prominences: 48.01 Hz and 5.42st vs. TOP-prominences: 36.14 Hz and 4.16st); instead, in CIPPS, the *f0sd* values are lower for the Comment (42.02 Hz and 5.01st) than for the Topic (56.29 Hz and 6.31st).^26^

Although the reason for this finding in schizophrenic patients must still be investigated, it is worth noticing that the recorded qualitatively higher *f0sd* is consistent with previous studies on other pathologies (depression in Silva et al., 2021; mutational falsetto, laryngeal carcinoma, and vocal cord polyps in Li et al., 2021).

#### 3.3.3. emph

Regarding COM-prominences, the *emph* is 2.02 dB in CIPPS and 3.36 dB in SAMP(100). Thus, the schizophrenic speakers put less vocal effort than the non-pathological in producing the prominences bearing the illocution, in correlation with lower values of *f0mean*.

No particular differences, instead, are identified for TOP-prominences between the two groups: values in CIPPS (3.97 dB) are similar to those in SAMP(100) (3.03 dB).^27^

In other words, the performance of the illocution results in an attitude of acoustic “weakness,” flattening, and less effort is recorded. The datum is even more relevant, considering that this does not regard TOP-prominence. Therefore, a possible correlation with the monotony effect seems to be relative specifically to COM-prominence (Compton et al., 2018).

#### 3.3.4. cvint

For our goal, the coefficient of intensity variation (*cvint*) is more reliable than the direct intensity measurement since, in our corpora, neither the distance from the microphone nor the angle between the microphone and the speaker’s mouth was fixed, so altering the recorded intensity.

Again, given the monotony perceived in pathological speech, the starting hypothesis is that in CIPPS, *cvint* values are lower than those of the control group.

The data confirms expectations: for COM-prominences, the *cvint* is three times lower in CIPPS (2.78) than in non-pathological speech (6.72), while for TOP-prominences the difference is reduced (3.44 vs. 5.32).^28^

## 4. Discussion

The analysis conducted on CIPPS and its comparison with the control group highlights the peculiarity of schizophrenic speech compared to the threshold values recorded in non-pathological trends of spontaneous dialogs in the various linguistic domains considered in this research. Results can be summarized as follows.

Regarding the structure of the TS, from a qualitative point of view, utterances are shorter in terms of MLU and less articulated in schizophrenic patients, but the intersubjective variability is high. All patients, however, prefer delocalized post-nuclear information units (Appendix) and, as expected, a low number of stanzas; thus, the speech is structured in smaller chunks and less organized from an informational point of view.

Moreover, the fluency is interrupted by an atypical number of very long pauses (1–20 s). Pauses do not occur in connection to the locutionary programming inside the utterance but mostly regard its pragmatic conception with a substantial turn-taking delay (cf. Alpert et al., 2000; Lucarini et al., 2022).

The quantity of retracing phenomena highlights patients’ difficulty in programming the locution; according to our findings, the incidence of retracing rises specifically when the discourse is structured in complex utterances and stanzas. Retracing chains turn out to be associated with the disease in a more substantial way than single episodes.

Lastly, the analysis of prominences brought about the following findings:

- In CIPPS, the nuclear part of the COM unit is characterized by lower values of *f0mean*, *emph*, and *cvint,* while the *f0sd* is higher. The prosodic parameters reflect an attitude of acoustic “weakness” of the performance of the illocution, which can be one of the causes of the perceived monotony. The lowering of the above values suggests an impairment in dealing with the variability of the illocutions and a lack of engagement in the communicative events (cf. Pellet-Rostaing et al., 2023).
- On the other hand, the measured values of the nuclear part of the TOP are lower for *cvint*, similar for *f0mean* but higher for *f0sd* and *emph* concerning the controls. This suggests that schizophrenic speech is characterized by greater effort when defining the Topic, i.e., the domain of illocutionary force.
- The differences between COM- and TOP-prominences highlight the relevance of dividing the analysis for the two information units. Beyond the previous differences, COM- and TOP-prominences record a high variation between them for *f0mean*, *f0sd*, and *emph* in CIPPS, which is not found in SAMP(100). Moreover, in CIPPS, TOP-prominences record higher values than the COM according to all the detected parameters. In contrast, the control group follows the opposite trend, except for the *f0mean,* which varies in a limited manner. The different attitudes toward the performance of the two units could be an index of schizophrenic atypia.

All the findings have been processed to investigate whether the results have statistical relevance. The Kruskal-Wallis test for not normally distributed data has been used, and data for each patient have been compared to the control groups, although without reporting significant differences. Our sample sizes are not conducive to inferential statistics due to the preference for a corpus-based methodology, which represents spontaneous speech variability rather than verifying the behavior of two populations facing the same task.

The results discussed here shall be understood as a qualitative description and shed light on the specificity of schizophrenic linguistic profiles, which still need more extensive studies. Moreover, one implication of our analyses is to suggest future directions of investigation where the tests above highlight differences between the datasets. For this purpose, designing larger and statistically sound samplings will be useful.

In sum, the terminated sequences of CIPPS appear generally short, lacking in informative articulation, often interrupted by disfluency phenomena, and prosodically flat when performing the illocutionary pragmatic activity.

Thanks to the L-AcT approach, it has been possible to divide the linguistic analysis into distinct levels, allowing the highlighting of the specific features for each level responsible for the perceived monotony of schizophrenic speech.

## Data availability statement

Publicly available datasets were analyzed in this study. This data can be found here: http://corpus.lablita.it/?locale=en.

## Ethics statement

Ethical approval was not required for the study involving humans in accordance with the local legislation and institutional requirements. Written informed consent to participate in this study was not required from the participants or the participants’ legal guardians/next of kin in accordance with the national legislation and the institutional requirements.

## Author contributions

VS wrote sections 1, 2.2, and 3.1. ST wrote sections 2.1, 3.2, and 3.3. MM supervised the research. VS, ST, and MM wrote and conceived the discussion section together. All the authors contributed to the conception and design of the study and have approved the final version of the manuscript.

## Footnotes

^1^Cf. Overall and Gorham (1962) for the Brief Psychiatric Rating Scale “BPRS” with 16 items; Andreasen (1979) for the Scale for the Assessment of Thought, Language, and Communication Disorders “TLC”; Andreasen (1982) for the Scale for the Assessment of Negative Symptoms “SANS”; Andreasen (1986) for the Scale for the Assessment of Positive Symptoms “SAPS.”

^2^Patients have been recruited in collaboration with Doctor Pastore at “Scuola Sperimentale per la Formazione alla Psicoterapia e alla Ricerca nel Campo delle Scienze Umane Applicate” of ASL NA1 of Naples and Prof Albano Leoni at CIRASS in 2005. All the participants are recorded with informed written consent. The source audio files are publicly available on CD.

^3^The source audio files are available on http://corpus.lablita.it.

^4^The psychopathological description of each patient does not comprise standard testing, which is not available from published materials.

^5^See Berrios (1996) for the background of the terminology. In particular, the term Wahnstimmung, also called “Delusional mood” (Conrad, 1958; Mishara, 2010), is a prodromal feature of an impending psychotic illness in which the patient has the feeling that “something is in the air,” a delusion of catastrophe in the world. The prevalence of Wahnstimmung in schizophrenia spectrum disorder was recently described as between 1 and 8% (Blom, 2015).

^6^The speakers of SAMP are named “cami,” “fale,” “pell,” and “vefa.”

^7^They find a negative association between average pause duration and “existential reorientation,” which refers to a fundamental rearrangement concerning patients’ general metaphysical worldview and/or hierarchy of values, projects, and interests.

^8^Pauses “after” the turn are excluded because they are influenced by the attitude of the doctor in the communicative context.

^9^Excluding the retracted tokens (see below).

^10^Cf. Moneglia (2005, p. 58-59) for a description of MLU variations across language contexts in Italian non-pathological speech, which is consistent with these data for what regards monologs.

^11^Cutting the Sample following the duration parameter highlights the peculiarity of A’s behavior in the communicative exchange with the doctor. For him, the number of terminated sequences in 15′ of recordings is the lowest of the Sample, so he covers only 1/10 of the CIPPS excerpts here commented. This is reflected in the massive presence of pauses (see 3.2.1).

^12^Similar analyses have been conducted on schizophrenic speech in Brazilian Portuguese, see Rocha et al. (2022).

^13^It should be noted that in the work mentioned above, data is measured in relation to the number of terminated sequences per communicative event (dialog/conversation/monolog); since our data here are measured speaker by speaker, the numbers do not consider the interlocutor’s turns. Hence, we expect the percentage of stanzas to be higher than the reference value reported in Saccone (2022).

^14^For pauses and retracing phenomena, statistical tests were not applied because of data aggregation strategy.

^15^We carried out an agreement test between annotators, resulting in a rate of 0.85. The test agreement has been made on a sample of D. On the basis of the silent/sounding detection, we observed the manually verified boundaries comparing starting (t-min) and ending (t-max) times of silences. We adopted a fluctuation range of 150 ms, based on the minimum chosen threshold.

^16^Cf. Duez (1985) and Giannini (2008) for silences >180 ms.

^17^For these first preliminary analyses, the comparison group is the CORCON.

^18^See Cresti et al. (forthcoming) for a more detailed description.

^19^Only for one speaker of SAMP the values do reach the average of CIPPS.

^20^For this analysis, the comparison group is the CORCON.

^21^Parallel works are in progress at the LEEL lab of UFMG of Belo Horizonte, under the supervision of Tommaso Raso and Bruno Rocha.

^22^The part that precedes (or, rarely, follows) the prominence (*preparation*/*tail*) is also isolated for future works.

^23^Parameters of the script have been settled for each audio file according to the f0 range of the speaker.

^24^The parameters related to the f0 are calculated both in Hertz and in semitones. The values in Hertz show the absolute number of vibrations of the vocal cords in one second, while the ones in semitones, being a logarithmic transformation, indicate how the auditory system processes the vibrations (Barbosa, 2019) and therefore better reflects perceptual differences between frequencies.

^25^COM-prominence, values in Hz: A: value of *p* = 0.477; B: value of *p* = 0.282; C: value of *p* = 0.449; D: value of *p* = 0.545. COM-prominence, values in st: A: value of *p* = 0.09; B: value of *p* = 0.404; C: value of *p* = 0.901; D: value of *p* = 0.412. TOP-prominence, values in Hz: A: value of *p* = 0.33; B: value of *p* = 0.821; C: value of *p* = 0.391; D: value of *p* = 0.367. TOP-prominence, values in st: A: value of *p* = 0.306; B: value of *p* = 0.578; C: value of *p* = 0.375; D: value of *p* = 0.367.

^26^COM-prominence, values in Hz: A: value of *p* = 0.479; B: value of *p* = 0.479; C: value of *p* = 0.477; D: value of *p* = 0.477. COM-prominence, values in st: A: value of *p* = 0.451; B: value of *p* = 0.431; C: value of *p* = 0.366; D: value of *p* = 0.575. TOP-prominence, values in Hz: A: value of *p* = 0.33; B: value of *p* = 0.821; C: value of *p* = 0.391; D: value of *p* = 0.367. TOP-prominence, values in st: A: value of p = 0.; B: value of p = 0.; C: value of p = 0.; D: value of *p* = 0.

^27^COM-prominence: A: value of *p* = 0.988; B: value of *p* = 0.118; C: value of *p* = 0.752; D: value of *p* = 0.741. TOP-prominence: A: value of *p* = 0.423; B: value of p = 0.4; C: value of *p* = 0.481; D: value of p = 0.367.

^28^COM-prominence: A: value of *p* = 0.787; B: value of *p* = 0.368; C: value of *p* = 0.145; D: value of *p* = 0.866. TOP-prominence: A: value of *p* = 0.306; B: value of *p* = 0.966; C: value of *p* = 0.795; D: value of *p* = 0.479.
